# Comparison of population characteristics and clinical outcomes of patients with type B aortic dissection or aortic intramural hematoma underwent thoracic endovascular aortic repair: a propensity score-matched analysis

**DOI:** 10.1186/s13019-023-02280-8

**Published:** 2023-05-11

**Authors:** Nan Zhang, Tian-shu Xu, Tie-nan Zhou, Lei Zhang, Xiao-zeng Wang, Ying Min

**Affiliations:** 1 Department of Cardiology and Cardiovascular Research Institute, General Hospital of Northern Theater Command, Shenyang, 110016 Liaoning China; 2 China Medical University, Shenyang, 110122 Liaoning China

**Keywords:** Acute type B aortic intramural hematoma, Acute type B aortic dissection, Thoracic endovascular aortic repair

## Abstract

**Backgrounds:**

Survival and aortic-related adverse events after thoracic endovascular aortic repair (TEVAR) for aortic intramural hematoma (IMH) and aortic dissection (AD) are controversial. We aimed to assess the preoperative characteristics and to evaluate TEVAR outcomes of acute type B IMH and AD.

**Methods:**

Between June 2002 and May 2021, 83 patients with acute type B IMH and 755 patients with acute type B AD underwent TEVAR at the General Hospital of Northern Theater Command. We retrospectively analyzed data from these patients, including clinical characteristics and follow-up outcomes.

**Results:**

The patients with IMH were significantly older than the ones with AD (P < 0.001). Diabetes mellitus (P = 0.035) and ischemic cerebrovascular disease (P = 0.017) were more common in the IMH group than in the AD group. The results demonstrated a less long-term aortic-related death-free survival rate in the IMH group than the AD group for all the patients (P = 0.014) and the matched patients (P = 0.027). It also presents a lower long-term overall survival rate (P = 0.047) and aortic-related event-free rate (P = 0.048) in the IMH group than in the matched patients.

**Conclusions:**

Compared with AD patients, patients with IMH who underwent TEVAR had a worse long-term outcome of aortic-related survival in all and matched patients.

## Introduction

Intramural aortic hematoma (IMH) is a life-threatening aortic disorder that varies from acute aortic dissection (AD) in that it is a contained aortic wall hematoma with intramural hemorrhage that occurs without the formation of an intimal flap [1]. Two main pathophysiologic mechanisms have been linked to the occurrence of IMH. One is aortic vasa vasorum rupture, which causes a hematoma in the media of the aorta wall without intimal disruption [2]. Another reason is thought to be the intimal disruptions. Recent studies have shown the presence of various sizes of focal intimal disruption (FID) at the onset of IMH, which may be related to the pathogenesis and clinical outcome of IMH [3, 4]. The prognosis of IMH is unpredictable: some IMHs regress [5] and are completely resorbed [6], while others progress to classic AD [6], aortic rupture [7], or aneurysmal dilation [8]. According to Stanford’s classification, IMH can be classified as type A (involving ascending aorta) and type B (not involving ascending aorta) IMH. Patients with type A IMH have benefited from the operations and have had greater survival rates in recent years [9]. When compared to type AAD, patients with type A IMH exhibited lower surgical mortality and a superior 5-year survival rate [9]. However, the management of patients with type B IMH is still controversial. A study showed that the acute phase mortality results of medical therapy were lower than that in TEVAR [10]. The other two studies, however, advocated TEVAR as the treatment of choice for people with type B IMH because medical treatment is ineffective [11, 12]. Adam etc. found that TEVAR was associated with a lower risk of dissection and a lower risk of rupture in type B IMH patients than medical therapy [10]. TEVAR is being utilized more actively to improve long-term survival, reintervention rates (surgical or interventional treatment), and favorable aortic remodeling. However, it was unclear whether TEVAR was useful to patients with complicated type B IMH. TEVAR is a Class IIa recommendation for complicated type B IMH, but only at a Level C evidence level [13]. As a result, we aim to investigate the preoperative characteristics and long-term outcomes of patients with type B IMH and type B AD who underwent TEVAR in this study.

## Materials and methods

### Patient population

This retrospective study was approved by the Ethics Committee of General Hospital of Northern Theater Command [Y (2021) 073, 2021/9/2)], and a waiver of informed consent was obtained. There were 1967 consecutive patients with the aortic disease (< 14 days) who were admitted to the General Hospital of Northern Theater Command between June 2002 and May 2021. We excluded type A AD (n = 529), pseudoaneurysm (n = 59), ascending aortic aneurysm (n = 47), thoracic aortic aneurysm (n = 39), abdominal aortic aneurysm (n = 78), Marfan syndrome (n = 42), traumatic dissection (n = 28), type B AD and IMH treated with medical therapy only (n = 137), surgical (n = 32), and missing medical features (n = 138). Finally, 83 patients with type B IMH and 755 patients with type B AD who underwent TEVAR were enrolled in this study. Briefly, once type B IMH patients were diagnosed by computed tomographic angiography (CTA) as intramural hematoma, they were reexamined by CTA 1–2 weeks later. If the type B IMH was found to be transformed into IMH with FID, type B AD, or impending thoracic aortic rupture (localized progressive thickening of IMH with pleural effusion), TEVAR would be performed.

### Data collection and definitions

The following information was collected from patient medical records: age, sex, chest pain, limb pain, conscious disorder, smoking, drinking alcohol, hypertension, gout, myocardial ischemia, diabetes mellitus (DM), hemorrhagic cerebrovascular disease (HCD), ischemic cerebrovascular disease (ICD), pulmonary disease, family history of hypertension, family history of cerebrovascular disease (CED), family history of aneurysm or AD, family history of coronary artery disease (CAD), and valve abnormality (Table 1). Records for the study population were reviewed, including procedural details and operative data such as operative death, endoleak, new fever and complications (Table 2).

**Table 1:** Preoperative characteristics of the patients

Variables	All patients			Propensity-matched pairs		
	IMH group (n = 83)	AD group (n = 755)	*P* value	IMH group (n = 80)	AD group (n = 80)	*P* value
Age (y; mean ± SD)	61.17$\pm$10.24	54.12$\pm$11.64	≺0.001	60.74$\pm$10.14	58.39$\pm$11.49	0.173
Male	61(73.5%)	609(80.7)	0.122	59(73.8%)	56(70.0%)	0.598
Chest pain	77(92.8%)	704(93.2%)	0.8716	74(92.5%)	75(93.8%)	0.755
Limb pain	2(2.4%)	17(2.3%)	0.927	2(2.5%)	1(1.3%)	0.560
Conscious disorder	5(6.0%)	33(4.4%)	0.492	5(6.3%)	5(6.3%)	0.157
Smoking	55(66.3%)	461(61.1%)	0.355	53(66.3)	47(58.8%)	0.327
Drinking alcohol	39(47.0%)	315(41.7%)	0.357	38(47.5%)	29(36.3%)	0.149
Hypertension	68(81.9%)	639(84.6%)	0.519	65(81.3%)	73(91.3%)	0.066
Gout	1(1.2%)	10(1.3%)	0.928	1(1.3%)	1(1.3%)	1.000
Myocardial ischemia	12(14.5%)	126(16.7%)	0.603	11(13.8%)	18(22.5%)	0.151
Diabetes mellitus	9(10.8%)	39(5.2)	0.035	8(10.0%)	9(11.3%)	0.798
HCD	1(1.2%)	13(1.7%)	0.727	1(1.3%)	2(2.5%)	0.560
ICD	14(17.3%)	67(9.0%)	0.017	14(17.5%)	20(25.0%)	0.246
Pulmonary disease	1(1.2%)	39(5.2%)	0.108	1(1.3%)	2(2.5%)	0.560
Family history of hypertension	8(9.6%)	101(13.4%)	0.336	8(10.0%)	12(15.0%)	0.339
Family history of CED	4(4.8%)	23(3.0%)	0.385	4(5.0%)	4(5.0%)	1.000
Family history of aneurysm or AD	0(0.0%)	4(0.5%)	0.506	0(0.0%)	0(0.0%)	NA
Family history of CAD	1(1.2%)	11(1.5%)	0.854	1(1.3%)	0(0.0%)	0.316
Valve abnormality	27(32.5%)	262(34.7%)	0.693	27(33.8%)	25(31.3%)	0.736

HCD: hemorrhagic cerebrovascular disease; ICD: ischemic cerebrovascular disease; CED: cerebrovascular disease; AD: acute dissection; CAD: coronary artery disease; NA: not applicable

**Table 2:** In-hospital outcomes of TEVAR in patients

Variables	All patients				Propensity-matched pairs		
	IMH group (n = 83)	AD group (n = 755)	*P* value		IMH group (n = 80)	AD group (n = 80)	*P* value
Operative death	0(0.0%)	10(1.3%)	0.292		0(0.0%)	1(1.3%)	0.316
New-onset fever	15(19.0%)	334(45.0%)	< 0.001		15(19.7%)	29(36.7%)	0.019
White blood cell ($\times$10^9^/L)	10.57$\pm$2.709	11.92$\pm$3.315	< 0.001		10.68$\pm$2.690	11.96$\pm$2.983	0.006
Hypersensitive C-reactive protein (mg/L)	66.38$\pm$60.733	92.10$\pm$75.037	0.007		66.91$\pm$61.995	96.74$\pm$82.407	0.034
Complications	10(12.0%)	93 (12.3%)	0.943		10(12.5%)	7(8.8%)	0.442
Cerebral hemorrhage	0(0.0%)	2(0.3%)	0.639		0(0.0%)	0(0.0%)	NA
Cerebral infarction	0(0.0%)	6(0.8%)	0.415		0(0.0%)	0(0.0%)	NA
Myocardial injury or myocardial infarction	0(0.0%)	10(1.3%)	0.292		0(0.0%)	0(0.0%)	NA
Arrhythmia	1(1.2%)	14(1.9%)	0.672		1(1.3%)	0(0.0%)	0.316
Heart failure	0(0.0%)	1(0.1)	0.740		0(0.0%)	1(1.3%)	0.316
Heart arrest	0(0.0%)	3(0.4%)	0.565		0(0.0%)	0(0.0%)	NA
Mesenteric injury	0(0.0%)	2(0.3%)	0.639		0(0.0%)	0(0.0%)	NA
Acute renal failure	1(1.2%)	25(3.3%)	0.293		1(1.3%)	2(2.5%)	0.560
Abnormal liver function	2(2.4%)	15(2.0%)	0.795		2(2.5%)	1(1.3%)	0.560
Shock	0(0.0%)	8(1.1%)	0.346		0(0.0%)	1(1.3%)	0.316
Cardiac tamponade	0(0.0%)	2(0.3%)	0.639		0(0.0%)	0(0.0%)	NA
Paraplegia	0(0.0%)	1(0.1%)	0.740		0(0.0%)	0(0.0%)	NA
Limb thrombus	0(0.0%)	6(0.8%)	0.415		0(0.0%)	1(1.3%)	0.316
Abnormal lower extremities	0(0.0%)	13(1.7%)	0.228		0(0.0%)	2(2.5%)	0.155
Infectious disease	7(8.4%)	32(4.2%)	0.085		7(8.8%)	3(3.8%)	0.191

Complications contain cerebral hemorrhage, cerebral infarction, myocardial injury or myocardial infarction, arrhythmia, heart failure, heart arrest, mesenteric injury, acute renal failure, abnormal liver function, shock, cardiac tamponade, paraplegia, limb thrombus, abnormal lower extremities and infectious disease. b) NA, not applicable

Type B IMH was defined as the hematoma on the aortic wall, absence of an intimal flap or tear on CTA, and only involving the descending aorta. Complicated Type B IMH was described as patients with persistent or recurrent pain, uncontrolled hypertension despite comprehensive medical therapy, hematoma or aorta enlargement, malperfusion, intimal disruption, or impending rupture [13, 14]. One patient might present with more than one of these symptoms at the same time. The aortic enlargement was defined as an increase in maximal diameter in the descending aorta. Type B AD was characterized by CTA as a rupture of the aortic intima, with blood flow in the false lumen penetrating the media of the descending aortic wall without the involvement of the ascending aorta. And all AD patients enrolled were considered to be complicated AD for its severely persistent or recurrent pain. Smokers were classified as those who smoked at that point or had a previous smoking history; non-smokers were defined as those who had never smoked in their lives. Alcoholics were the people who drank alcohol at that time or had been alcoholics previously and non-alcoholics were described as the people who had never drunk alcohol in their lives. Valve abnormalities were defined as aortic, mitral, and tricuspid regurgitation by echocardiography. In-hospital death was defined as the death after TEVAR and occurred in the hospital. The 30-day death was defined as the death within 30 days after being charged from the hospital. A new-onset fever was defined as the patient’s body temperature being higher than or equal to 37.5 ℃ after TEVAR. Complications include cerebral hemorrhage, cerebral infarction, myocardial injury, mesenteric injury, acute renal failure, abnormal liver function, shock, cardiac tamponade, paraplegia, and lower limb abnormalities, of which symptoms include chill, pain, whiteness, impaired mobility, or weak pulse.

### Treatment strategy

All patients were treated with initially medical therapy immediately to control pain and blood pressure under intensive monitoring. TEVAR procedures were performed for all type B AD patients and complicated type B IMH [14]. All type B AD patients and complicated type B IMH received an emergent TEVAR after sufficient pain-control, anti-hypertension therapy, which could usually be achieved within 3–5 days after admission. And for those IMH patients with initially uncomplicated conditions, we would monitor them with follow-up procedures to detect if they converted to a complicated IMH and thus to determine if they needed to receive an elective TEVAR. TEVAR procedure has been described in previous literature in detail [15]. In brief, general or epidural anesthesia was administered in the cardiac catheter room. Aortic angiography was performed by using a catheter inserted into the left radial artery to assess the location, morphology, and extent of the dissection or the intramural hematoma. According to the measured data, the operator accurately chose the specifications, types, and numbers of the covered stents. The dimensions of the proximal and distal landing zones were at least 20 mm (according to the instructions for most devices) and were evaluated preoperatively using three-dimensional reconstruction software and centerline measurements. At the same position, the outer diameter of the covered stent exceeded 10–20% of the real lumen inner diameter. To ensure that the covered stent system was delivered into the rupture site of descending AD through the abdominal aorta along the wire in the true lumen, the operator used a femoral (or external iliac) artery incision as the surgical approach. The aortic angiography was utilized to determine the dissection and hematoma sealing effect after the covered stent was released.

### Patient’s follow-up and study endpoints

Patients in both groups were followed up at 1, 6, and 12 months after TEVAR; and then annually. A CTA was routinely performed in asymptomatic patients within 6–12 months or performed when patients suffering from the symptoms such as chest pain or abdominal pain. Follow-up data were collected by reviewing telephone call records, outpatient visit records, and readmission records. The primary endpoint was freedom from aortic-related death. Secondary endpoints included overall survival and freedom from adverse aortic-related events, which included aortic-related death, recurrence of dissection, stent endoleak, distal ulcer, stent thrombosis, retrograde tear, and aortic rupture.

### Statistical analysis

SPSS 26.0 was used for statistical analysis. (SPSS Inc., Chicago, IL). Continuous variables are shown as the mean ± standard deviation (SD); t-test was used to analyze the differences. Categorical variables are presented as a frequency; differences were determined by using the Chi-square test. Univariate analyses were carried out using the t-test for continuous variables and Fisher’s exact test for categoric variables. Kaplan–Meier method was used to perform survival analysis. Differences in survival analysis were compared by the log-rank test. A 1: 1 propensity score matching (PSM) analysis was performed to reduce potential selection bias with the following covariates: age, sex, chest pain, limb pain, coma, smoking, drinking alcohol, hypertension, gout, myocardial malperfusion, DM, HCD, ICD, pulmonary disease, family history of hypertension, family history of CED, family history of aneurysm or AD, family history of CAD, and valve abnormality. Propensity score matching yielded a cohort of 80 patients in the IMH group and 80 in the AD group. A two-tailed *P* value less than 0.05 was considered statistically different.

## Result

### Preoperative characteristics of all patients

Table 1 shows the characteristics of the patients in both groups. The IMH group was significantly older age than the AD group (61.17$\pm$10.24 years vs. 54.12 $\pm$11.64 years; *P* < 0.000). DM (10.8% vs. 5.2%, *P* = 0.035) and ICD (17.3% vs. 9.0%, *P* = 0.017) were more frequently observed in the IMH group than in the AD group. No significant differences were observed between the two cohorts in terms of sex, chest pain, limb pain, conscious disorder, smoking, drinking alcohol, hypertension, gout, myocardial ischemia, HCD, pulmonary disease, family history of diseases (CED, aneurysm or AD and CAD, respectively) and valve abnormality (*P* > 0.05).

### Operative outcomes of TEVAR in all and propensity score-matched patients

Table 2 displays the operational results. In all patients, the incidence of new-onset fever was significantly lower in the IMH group than in the AD group (19.0% vs. 45.0%, *P* = 0.000), and there was no significant difference in postoperative complications (12.0% vs. 12.3%, *P* = 0.943) or in-hospital death (0.0% vs. 1.3%, *P* = 0.292) between the two groups. In paired patients, the new-onset fever rate after TEVAR in the IMH group remained lower than in the AD group (19.7% vs. 36.7%, *P* = 0.019, Table 2), and we found no significant differences in in-hospital deaths (0.0% vs. 1.3%, *P* = 0.316) or complications (12.5% vs. 8.8%, *P* = 0.442) between the two groups (Table 2).

### Thirty-day survival and freedom from aortic-related adverse events

**Fig. 1:**
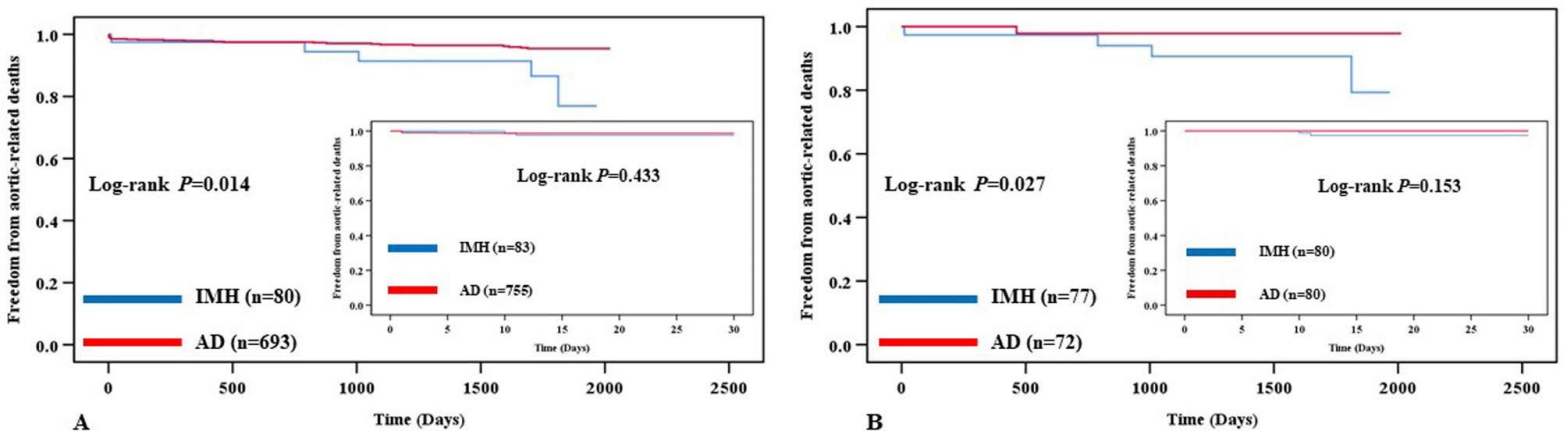
Kaplan–Meier survival analysis from 30-day and Long-term outcome for aortic-related death-free rates in all (Fig. 1. A) and in the matched patients (Fig. 1. B)

**Fig. 2:**
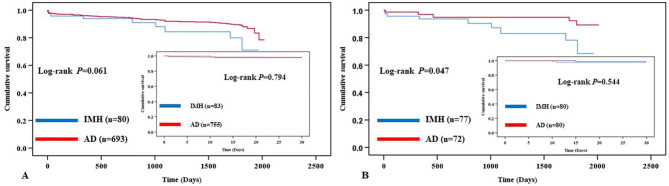
Kaplan–Meier survival analysis from 30-day and Long-term outcome for freedom from any cause of death in all (Fig. 2. A) and in the matched patients (Fig. 2. B)

**Fig. 3:**
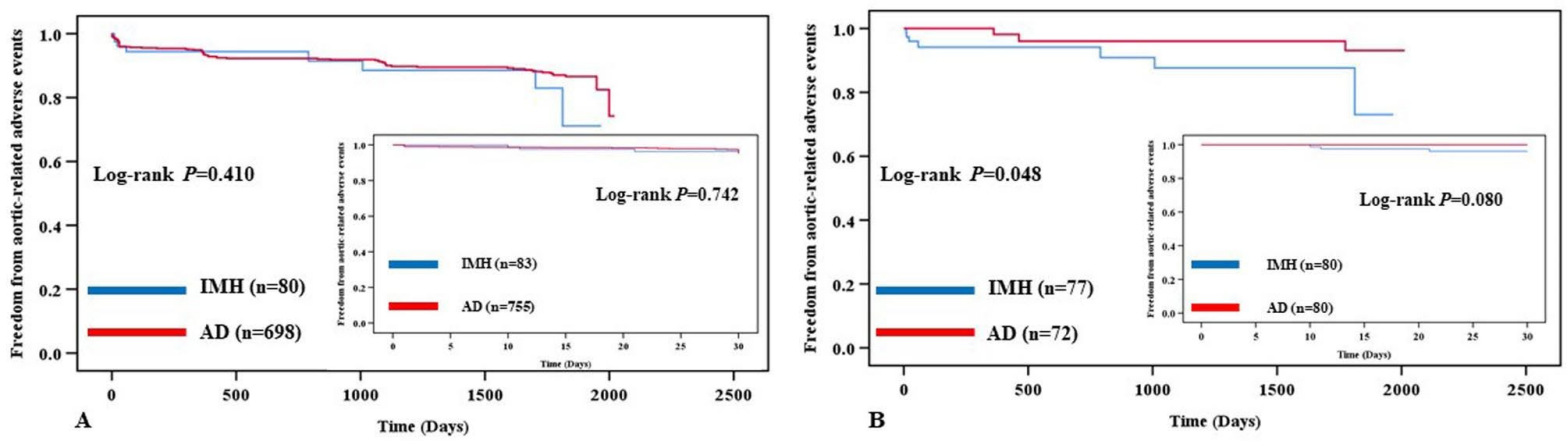
A, Kaplan–Meier analysis from 30-day and Long-term outcome for aortic-related event-free rates in all patients. B, Kaplan–Meier analysis from 30-day and Long-term outcomes for aortic-related event-free rates with 95% confidence limits in the matched patients

All patients (100%) were followed up for 30 days. There were only 2 aortic-related deaths and no other deaths within 30 days in the IMH group. While 15 all-cause deaths occurred in the AD group, of which, 10 were aortic rupture, 2 died of multi-organ dysfunction syndrome (MODS), 1 died of cancer, 1 died of brain hemorrhage, and 1 died of uremia. The log-rank test revealed similar 30-day freedom from aortic-related deaths in the IMH group versus the AD group for all patients (97.6% vs. 98.7%, P = 0.433) (Fig. 1, A). The 30-day survivals were not significantly different between the two groups (97.6% vs. 98.0%, *P =* 0.794) (Fig. 2, A). There was no significant difference in 30-day freedom from aortic-related adverse events (96.4% vs. 95.8%, *P =* 0.742) (Fig. 3, A). In the propensity-matched patients, there weren’t statistical differences at the 30-day freedom from aortic-related death (97.5% vs. 100.0%, *P =* 0.153) (Fig. 1, B), overall survivals (97.5% vs. 98.8%, *P =* 0.544) (Fig. 2, B), and freedom from aortic-related adverse events (96.3% vs. 100.0%, *P* = 0.080) (Fig. 3, B).

### Long-term aortic-related mortality and all-cause mortality

The median follow-up period was 1189 days for the 773 patients, ranging from 540 days to 2021 days. During the long-term follow-up period, 9 patients in the IMH group died, including 6 aortic ruptures and 3 deaths from other causes (lung hemorrhage in 1 patient and unknown reason in 2 patients) in the IMH group. 65 patients died in the AD group, including 25 aortic ruptures, and 40 deaths with no relation to AD (brain-derived death in 12, cardiac death in 8, cancer in 7, sudden death in 3, MODS in 3, uremia in 1, leukemia in 1, respiratory failure in 1, and unknown reason in 4 patients).

As of 16 August 2021, the log-rank test revealed significant freedom from aortic-related mortality rates in the IMH group compared with the AD group for all the patients at the long-term outcome (92.5% vs. 96.4%, *P* = 0.014) (Fig. 1, A). Kaplan-Meier analysis estimated that the long-term outcome of overall survival of all patients were 88.8% in the IMH group, and 90.6% in the AD group (*P* = 0.061). In the propensity-matched patients, the log-rank test revealed significantly higher freedom from aortic-related mortality rate in the IMH group than in the AD group at long-term outcomes (93.5% vs. 98.6%, *P* = 0.027) (Fig. 1, B). Kaplan-Meier analysis also demonstrated that less survival rate of long-term outcomes in the IMH group than in the AD group in the matched patients (88.3% vs. 93.1%, *P* = 0.047) (Fig. 2, B).

### Long-term aortic-related adverse events

The median follow-up period was 1098 days for the 778 patients, ranging from 540 days to 2021 days. There were no statistical differences between the two groups, in terms of aortic-related death, recurrence of dissection, stent endoleak, distal ulcer, stent thrombosis, retrograde tear, and aortic rupture (Table 3). Kaplan-Meier analysis estimated that actuarial long-term aortic-related event-free rates were 90.0% in the IMH group, while 89.7% in the AD group, respectively (Fig. 3A). In unmatched patients, no significant differences in aortic-related event-free rates were found in the long-term follow-up (*P* = 0.410). However, in the propensity-matched patients, Kaplan-Meier analysis showed lower freedom from aortic-related adverse events in the IMH group than in the AD group in the long-term period (90.9% vs. 95.8%, *P* = 0.048).

**Table 3:** Long-term outcomes of adverse aortic-related events after TEVAR

Variables	All patients			Propensity-matched pairs		
	IMH group (n = 83)	AD group (n = 755)	*P* value	IMH group (n = 80)	AD group (n = 80)	*P* value
Aortic-related death	6(7.2%)	25(3.3%)	0.137	6(7.5%)	1(1.3%)	0.117
Aortic rupture	2(2.4%)	12(1.6%)	0.919	2(2.5%)	2(2.5%)	1.000
Dissection recurrence	1(1.2%)	20(2.6%)	0.668	1(1.3%)	1(1.3%)	1.000
Stent endoleak	0(0)	21(2.8%)	0.242	0(0)	0(0)	NA
Distal ulcer	0(0)	19(2.5%)	0.283	0(0)	0(0)	NA
Stent thrombosis	0(0)	3(0.4%)	1.000	0(0)	0(0)	NA
Retrograde tear	0(0)	8(1.1%)	1.000	0(0)	0(0)	NA
Overall events	9(10.8%)	108(14.3%)	0.388	9(11.3%)	4(5.0%)	0.148

Overall events including aortic-related death, aortic rupture, dissection recurrence, stent endoleak, distal ulcer, stent thrombosis, retrograde tear

## Discussion

This study suggested that patients with type B IMH were older than those with AD. DM and ICD were more frequently observed in the IMH group than in the AD group. Several studies have shown that AD happens in younger patients and more often in male patients than IMH [16, 17]. Smoking and hypertension are the known risk factors for aortic disease. A recent study reported that up to 70% of IMH patients had a history of hypertension [18]. 53.3% of patients with type B IMH had high blood pressure, and 60% of patients with type B IMH developed an aortic aneurysm at mid-term follow-up [18]. Moreover, hypertension and smoking may be more closely associated with acute IMH than acute AD [19]. Our results showed no significant differences between the two groups in terms of hypertension and smoking.

There is a lack of reports on TEVAR outcomes in a large sample of patients with type B IMH and AD [20]. Our research demonstrated similar 30-day survival and freedom from aortic-related adverse events after TEVAR in two groups according to the analysis of all patients or matched patients. Zhang and colleagues reported a 30-day mortality rate of 3.3% in the acute AD group [21]. An interdisciplinary consensus statement on the management of type B IMH and FID reviewed 18 publications and discovered a 30-day mortality rate of 4.6% after TEVAR for type B IMH [14]. Our study result was similar to the research described above.

Previous studies have reported no significant difference in long-term outcomes of drug therapy between type B IMH and type B AD, and a lack of a direct comparison of the long-term outcomes between type B IMH and type B AD cohorts after TEVAR [22]. Our study investigated the long-term outcome of TEVAR in patients with type B IMH compared with those with type B AD. In our study, the long-term outcome of freedom from aortic-related deaths was 92.5% compared to 96.4% in the AD group. It seems that patients with type B IMH after TEVAR had less long-term outcomes of aortic-related death-free survival compared with those with AD. This might be due to the lack of re-entry in the aortic wall in IMH, which could result in a greater tendency to aortic rupture [2]. Chen etc. found that aortic-related mortality was 2.5% in the TEVAR group of type B patients in the first year [23]. The cumulative freedom from aortic-related mortality of another study was 92.8% in the acute AD group after TEVAR at 3 years [21], similar to our study.

Our study displayed that long-term cumulated survivals in type B IMH were 88.8% and 90.6% in the AD group, respectively. A study reported that cumulative freedom from all-cause mortality was 89.5% at 3 years in acute complicated AD group [21]. Liu etc. found encouraging short-term and mid-term outcomes for Chinese patients with type B IMH with penetrating aortic ulcer (PAU) treated with TEVAR due to technical success rates and follow-up data [24]. They reported the 1-, 2-, and 5-year overall survival rates were 100%, 100%, and 96.1% in the type B PAU associated with IMH patients, respectively [24]. Compared to Liu’s results, our study had lower all-cause survival, which we considered due to the larger sample sizes and the different patient inclusion criteria.

A study reported that type B AD patients (2/14) needed less secondary intervention than IMH patients (2/8) [20]. We hypothesized that AD patients underwent secondary intervention for the stent distal anchor area of the original aortic intimal injury or the stent anchor area for the aortic junction. The intracapsular pressure of IMH was increased after the implantation of a self-expanding covered stent, so the stent edge was easily damaged. Consequently, an intimal rupture resulted in AD or pseudoaneurysm, where secondary TEVAR was needed. Patients underwent TEVAR with 0% rate of aortic-related adverse events during follow-up of 32 months ± 19 (range, 1–120 months) [13]. In Anna’s study, in patients with type B AD, the risk of an adverse event increased with aortic growth within the first six months after onset; and in IMH patients with risk of an adverse event was highest in the first year after onset and remained stable thereafter, which were inconsistent with ours. We speculated that it was due to the different treatment strategies adopted [25].

In our study, aortic-related survival and all-cause survival were lower in patients with IMH than in patients with AD in the long-term outcomes of all patients. And aortic-related survival, all-cause survival, and freedom from aortic adverse events were significantly lower in matched patients with IMH than in patients with AD. We considered lacking re-entry in IMH would be the main cause for this. Besides, based on previous literature [19] and clinical experience, we also speculated that the worse long-term outcomes for the survival of the IMH group might be related to the failure of hypertension control. We found that the awareness and control of hypertension were inadequate, and there were a few patients who didn’t take anti-hypertensive therapy at home during our follow-up. Some investigators believe that it is vital to control blood pressure to prevent aortic-associated complications [26]. Close follow-up is essential for patients with IMH. Clinicians should follow up with patients regularly and provide the necessary medical guidance.

There were several limitations in this study. Firstly, this was a retrospective study of single-centre registry data and some selection bias could not be eliminated. Secondly, we did not analyze the long-term clinical outcomes of patients after being discharged from hospital, which are worthy of further study. Furthermore, specific initial causes in complicated AD or IMH patients with multi-symptoms should be recorded in more detail to better present patients’ condition at admission.

## Conclusion

Our study revealed type B IMH patients who underwent TEVAR had worse long-term overall survival in matched groups. And AD group was observed a better aortic-related mortality-free rate than IMH group for unmatched and the matched groups at long-term period. Meanwhile, IMH showed a lower aortic-related event-free rates in the matched groups.

## Data Availability

All datas used during this study can be shared.

## Abbreviations

TEVAR: Thoracic endovascular aortic repair
IMH: Aortic intramural hematoma
AD: Aortic dissection
FID: Focal intimal disruption
PAU: Penetrating aortic ulcer
CTA: Computed tomographic angiography
DM: Diabetes mellitus
HCD: Hemorrhagic cerebrovascular disease
ICD: Ischemic cerebrovascular disease
CED: Cerebrovascular disease
CAD: Coronary artery disease
MODS: Multi-organ dysfunction syndrome
PIS: Post-implantation syndrome .
